# Alkaloids from Plants with Antimalarial Activity: A Review of Recent Studies

**DOI:** 10.1155/2020/8749083

**Published:** 2020-02-12

**Authors:** Philip F. Uzor

**Affiliations:** Department of Pharmaceutical and Medicinal Chemistry, University of Nigeria, 410001 Nsukka, Enugu State, Nigeria

## Abstract

Malaria is one of the major health problems in developing countries. The disease kills a large number of people every year and also affects financial status of many countries. Resistance of the plasmodium parasite, the causative agent, to the existing drugs, including chloroquine, mefloquine, and artemisinin based combination therapy (ACT), is a serious global issue in malaria treatment and control. This warrants an urgent quest for novel compounds, particularly from natural sources such as medicinal plants. Alkaloids have over the years been recognized as important phytoconstituents with interesting biological properties. In fact, the first successful antimalarial drug was quinine, an alkaloid, which was extracted from Cinchona tree. In the present review work, the alkaloids isolated and reported recently (2013 till 2019) to possess antimalarial activity are presented. Several classes of alkaloids, including terpenoidal, indole, bisindole, quinolone, and isoquinoline alkaloids, were identified with a promising antimalarial activity. It is hoped that the reports of the review work will spur further research into the structural modification and/or development of the interesting compounds as novel antimalarial drugs.

## 1. Introduction

Malaria is an extremely dangerous parasitic disease with ravaging effects in several parts of the world. The World Health Organization (WHO) estimate shows that approximately 3.3 billion people are living at risk places of malaria. Nearly 80% of cases and 90% of deaths are reported from sub-Saharan Africa and children under the age of 5 years and pregnant women are severely affected [1, 2]. In 2016, it was estimated that there were 216 million cases of malaria globally and 445,000 deaths due to malaria [3]. Five protozoan species of the genus *Plasmodium* (*Plasmodium falciparum*, *Plasmodium malariae, Plasmodium ovale*, *Plasmodium vivax*, and *P. knowlesi*) are responsible for human malaria, although the majority of malarial infections are caused by *P. falciparum* and *P. vivax.* While *P. vivax* is less dangerous but more widespread, *P. falciparum* is fatal and is predominant in Africa [1, 4].

**Figure 1:**
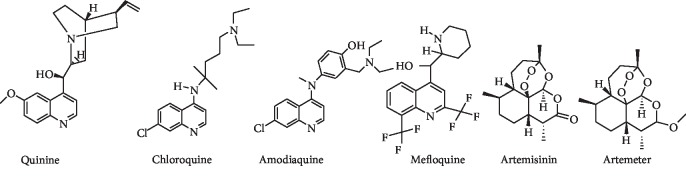
Structures of some antimalarial drugs.

Malaria has been treated with quinine, chloroquine, amodiaquine, mefloquine, and artemisinin derivatives (Figure 1), among other drugs. The alkaloidal drug, quinine, is the first antimalarial drug isolated from Cinchona bark. The drug is still quite useful in the treatment of multidrug-resistant malaria. Chloroquine, a 4-aminoquinoline, was developed in the 1940s as a synthetic derivative from quinine. It was effective, cheap, and less toxic and was the drug of choice for malarial treatment for decades; however, its use has been restricted in modern malaria therapy due to parasite resistance to the drug [5, 6]. Mefloquine is structurally related to quinine and has been introduced to treat chloroquine-resistant malaria, though its use is limited because of resistance and neuropsychiatric side effects [7]. Artemisinin is a natural endoperoxide isolated from sweet warm wood plant *Artemisia annua.* Artemisinin and its semisynthetic analogs artemether, artether, and artesunate are potent antimalarial agents especially used in the regions where the resistance has developed to other antimalarial agents. The WHO recommends the use of artemisinin analogs in combination with other drugs (ACT) for the treatment of malaria in order to control resistance. Unfortunately, there have been reports of parasite resistance to the ACT [8].

Given the development of resistance of the malarial parasites against many of the current treatment regimens, there has been urgent quest to identify new antimalarial chemotherapeutic agents from natural sources, particularly medicinal plants, in order to possibly avoid problems related to drug resistance [9–14]. This is due to the widespread use of plant materials in the treatment of malaria in many traditional medical practices together with the fact that plants were the sources of the two prominent antimalarial lead compounds, quinine and artemisinin.

Several classes of phytoconstituents are responsible for the antimalarial activity of plants including alkaloids, terpenes, steroids, and flavonoids. Alkaloids are considered as an important group exhibiting diverse biological activities, particularly antimalarial activity. They constitute an important class of structurally diversified compounds that are having the nitrogen atom in the heterocyclic ring and are derived from the amino acids [15]. Large numbers of alkaloids have been isolated from different plant sources and reported for their potent antimalarial activity, some of which have been previously reviewed up till the year 2012 [1, 13, 16–20]. However, more updates on the current research on alkaloids as potential antimalarial agents are needed. In the present review work, alkaloids from medicinal plants with antimalarial property which are reported recently from 2013 to 2019 are summarized. They are discussed in subclasses of alkaloids and the chemical structures of the newly reported compounds and those with antiplasmodial activity are shown in the figures according to their subclasses.

### 1.1. Terpenoidal and Steroidal Alkaloids

**Figure 2:**
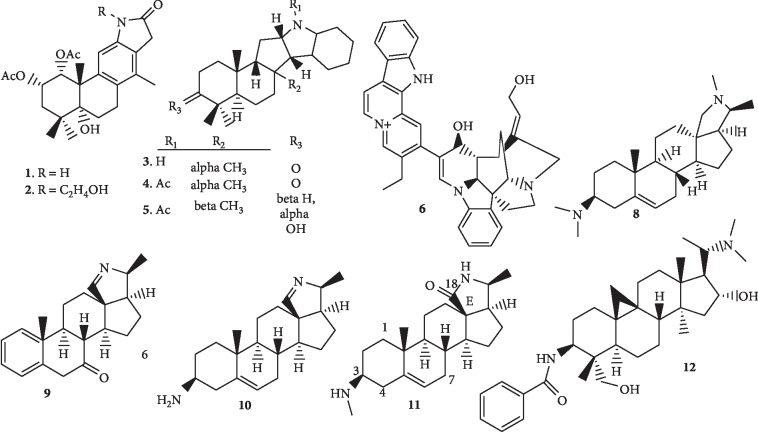
Structures of terpenoidal and steroidal alkaloids.

Several alkaloids having varying terpenoidal backbone, including the cassane-type diterpenes, indoloterpene, and bisindolomonoterpenic alkaloids, have been isolated recently from medicinal plants (*Caesalpinia minax*, *Polyalthia oliveri*, and *Strychnos nux-vomica*) and shown to possess good antiplasmodial activity (Figure 2).

Ma et al. [21] isolated two new diterpene alkaloids, caesalminines A (**1**) and B (**2**), possessing a tetracyclic cassane-type diterpenoid skeleton with *γ*-lactam ring from the seeds of *Caesalpinia minax* (Fabaceae). Compounds **1** and **2** exhibit antiplasmodial activity with IC_50_ values of 0.42 and 0.79 *μ*M, respectively. Substitution of the amino hydrogen of the diterpenoid (in compound **1)** with the alcoholic group (C_2_H_5_OH) produced a compound (**2**) with slightly reduced antiplasmodial activity. In another study, three new indolosesquiterpene alkaloids, 8*α*-polyveolinone (**3**), *N*-acetyl-8*α*-polyveolinone (**4**), and *N*-acetyl-polyveoline (**5**), together with three known compounds, were isolated from the stem bark of *Polyalthia oliveri* (Annonaceae) by Kouam et al. [22]. Compounds **4** and **5** exhibit moderate antiplasmodial activity against *P. falciparum* NF54 strain with low cytotoxicity on l myoblast (L6) cell line. In a related study of another plant in the same family, three bisindolomonoterpenic alkaloids, together with the known compound, strychnochrysine (**6**), were isolated from the stem bark of *Strychnos nux-vomica* L. (Loganiaceae). These longicaudatine-type alkaloids display antiplasmodial activity against both the chloroquine-resistant and chloroquine-sensitive strains. The most interesting was compound **6** with an IC_50_ value at around 10 *μ*M [23]. Muganza et al. [24] reported the isolation of two known indolosesquiterpene alkaloids, polyalthenol (**7**) and *N*-acetyl-polyveoline (**5**), from various parts of the tree *Greenwayodendron suaveolens* (Engl. & Diels) Verdc. (syn. *Polyalthia suaveolens* Engl. & Diels) (Annonaceae). The highest selectivity was observed for compound **5** against *P. falciparum* K1 (IC_50_ 2.8 *μ*M, selectivity index 10.9).

Alkaloids having steroidal nucleus have also been isolated and shown to possess antimalarial activity. Dua et al. [25] investigated the *in vitro* antimalarial and cytotoxic effects of the known compound, conessine (**8**), which was isolated from the plant *Holarrhena antidysenterica*. The four-day *in vivo* test was also used to test for the antimalarial activity against a chloroquine-sensitive *P. berghei* NK65 strain in BALB/c mice. Compound (**8**) shows *in vitro* antiplasmodial activity with its IC_50_ values of 1.9 *μ*g/ml and 1.3 *μ*g/ml in the schizont maturation and pLDH assays, respectively, and cytotoxicity IC_50_ of 14 *μ*g/ml. The compound also significantly reduces parasitaemia (88.95% parasite inhibition) in *P. berghei*-infected mice. In addition, the phytochemical investigation of an alkaloidal extract of the related plant *Holarrhena pubescens* roots led to the isolation of a new pregnene-type alkaloid, mokluangin D (**9**), together with nine known steroidal alkaloids. Two of the known compounds, irehline (**10**) and mokluangin A (**11**), show most potent antimalarial activity against *P. falciparum* K1 strain (a multidrug-resistant strain) with IC_50_ values of 1.2 and 2.0 *μ*M, respectively, and weak cytotoxic activity (27.7 and 30.6 *μ*M, respectively) against the NCI-H187 cell line. Five other compounds show moderate activity. Compound **9** was not tested. A study of the structure activity relationships in these compounds (illustrated with compound **11**) indicates that the C-3 amino group, the nature of the E ring, and the carbonyl group at C-18 are important for the activity against the *P. falciparum* K1 strain [26]. Furthermore, Pan et al. [27] reported the isolation of a known buxus alkaloid, *N*-3-benzoyldihydrocyclomicrophylline F (**12**), together with other compounds from *Buxus cochinchinensis* Pierre ex Gagnep. (Buxaceae). The buxus alkaloid (**12)** was found to show significant *in vitro* antimalarial activity against the drug-resistant Dd2 strain of *P. falciparum* with IC_50_ value of 2.07 *μ*M (cytotoxicity against HT-29 human carcinoma is 1.9 *μ*M and against NF-KB is >20).

### 1.2. Indole Alkaloids

**Figure 3:**
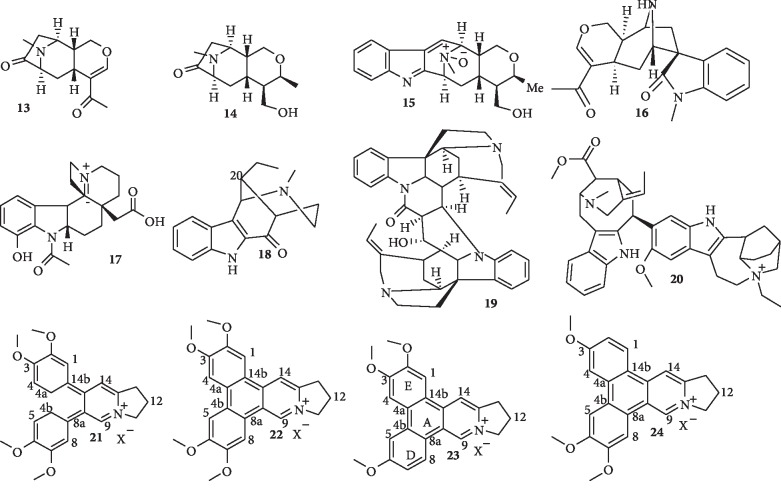
Structures of indole and related alkaloids with promising antiplasmodial activity.

A number of indole alkaloids (Figure 3) have been isolated recently and shown to possess potent antiplasmodial activity.

Chemical investigation of *Alstonia macrophylla* bark led to the isolation of two new nitrogenous derivatives, alstoniaphyllines A (**13**) and B (**14**), a new indole alkaloid, alstoniaphylline C (**15**), and eight known alkaloids including alstonisine (**16**). Compound **16** exhibits antiplasmodial activity against *P. falciparum*, with an IC_50_ value of 7.6 *μ*M [28]. Another new indole alkaloid, 12-hydroxy-*N*-acetyl-21(*N*)-dehydroplumeran-18-oic acid (**17**), and 11 known indole alkaloids including 20-epi-dasycarpidone (**18**) were isolated from *Aspidosperma ulei* Markgr. (Apocynaceae). Only compound **18** is active against multidrug-resistant K1 strain of *P. falciparum* with IC_50_ value of 16.7 *μ*M. None of these compounds exhibited toxicity to fibroblasts (IC_50_ > 50 *μ*g/mL) [29]. Also, Tchinda et al. [30] reported the isolation of a new bisindole alkaloid, strychnobaillonine (**19**), with original C-17-N-1′ and C-23-C-17′ junctions, in addition to three other known compounds from the roots of *Strychnos icaja*. Compound **19** shows potent activity against the chloroquine-sensitive 3D7 strain of *P. falciparum* with an IC_50_ value of 1.1 *μ*M. More recently, two new sarpagine-type indole alkaloids together with five known alkaloids, including 16-demethoxycarbonylvoacamine (**20**), were isolated from the bark of *Tabernaemontana macrocarpa* Jack. Compound **20** shows antiplasmodial activity against *P. falciparum* 3D7 and cytotoxic activity against human cell line, HepG2 cells [31].

### 1.3. Phenanthroindolizine Alkaloids

The structures of some phenanthroindolizine alkaloids with promising antiplasmodial activity are shown in Figure 3. Kubo et al. [32] isolated a new seco-phenanthroindolizine alkaloid, 4a,b-*seco*-dehydroantofine (**21**), and three known phenanthroindolizine alkaloids, dehydrotylophorine (**22**), dehydroantofine (**23**), and tylophoridicine D (**24**), from the twigs of *Ficus septica.* While compound **21** exhibits a moderate antimalarial activity against the 3D7 strain of *P. falciparum* with IC_50_ value 4.0 *μ*M, the known alkaloids (**22**–**24**) display strong activity with IC_50_ values of 0.42, 0.028, and 0.058 *μ*M, respectively. The cytotoxicities of the compounds (**21**–**24)** against mouse fibroblast cells L929 are >56 (SI ≥ 14), 8.2 (SI = 19.5), >55 (SI ≥ 1964), and >56 *μ*M (SI ≥ 966), respectively. Compounds **23** and **24** (particularly **23**) exhibit the highest selectivity against the malaria parasite. From the structure activity relationship, opening of ring A (present in compound **21**) possibly reduces antiplasmodial activity, while the four methoxy substituents at rings D and E (present in compound **23**) could be essential for activity.

### 1.4. Isoquinoline, Benzylisoquinoline, and Hasubanane Alkaloids

**Figure 4:**
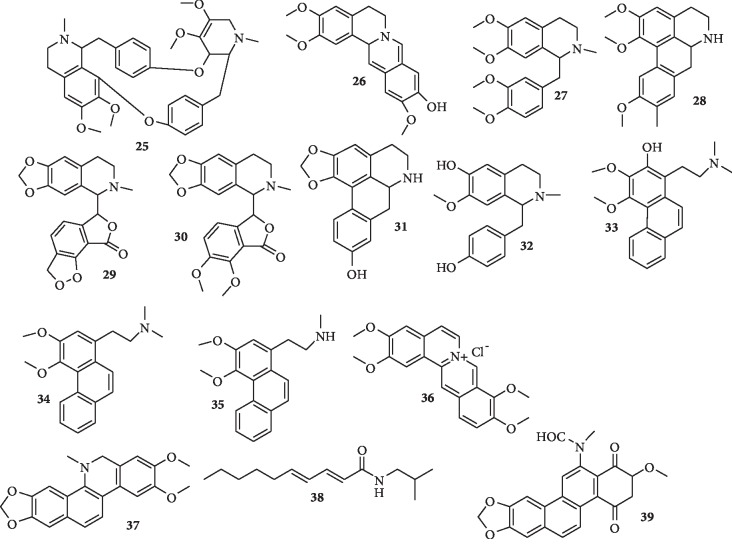
Structures of isoquinoline alkaloids.

Isoquinoline and related alkaloids with antiplasmodial activity have also been isolated. The structures of some of them are shown in Figure 4.

Fadaeinasab et al. [33] isolated seven isoquinoline alkaloids from the bark of *Actinodaphne macrophylla*. They display *in vitro* antiplasmodial activity against *P. falciparum* 3D7 with IC_50_ values ranging from 0.05 to 3.11 *μ*M for cycleanine (**25)**, 10-demethylxylopinine (**26**), reticuline (**27**), laurotetanine (**28**), bicuculine (**29**), *α*-hydrastine (**30**), and anolobine (**31**), respectively. Compound **26** was found to be the most active. Possibly, the fusion of the two isoquinoline rings (in compound **26**) enhances antiplasmodial activity. In another study, Nasrullah et al. [34] isolated four known alkaloids: (+)-*N*-methylisococlaurine (**32**), atherosperminine (**33**), 2-hydroxyathersperminine (**34**), and noratherosperminine (**35**) from the stem bark of *Cryptocarya nigra*. Compounds **32**, **33**, and **34** show strong antiplasmodial activity against a chloroquine-resistant strain of *P. falciparum* (K1 strain) with IC_50_ values of 5.40, 5.80, and 0.75 *μ*M, respectively. Compound **35** was not tested. The *N*,*N*-dimethyl groups of the ethylamine side chain (in compounds **33** and **34**) are, perhaps, essential for antiplasmodial activity. Malebo et al. [35] also isolated four pure alkaloids including palmatine (**36**) and mixtures of alkaloids from the leaves of *Annickia kummeriae*. The alkaloids exhibit low cytotoxicity (IC_50_ 30–>90 *μ*g/ml) and strong-to-moderate antiplasmodial activity (IC_50_ 0.08 ± 0.001–2.4 ± 0.642 *μ*g/ml, SI 1.5–1154). Compound **36** exhibits the highest activity against *P. falciparum* K1 (IC_50_ 0.080 ± 0.001 *μ*g/mL, selectivity index (SI) = 1154). Further, three known compounds, dihydronitidine (**37**), pellitorine (**38**), and heitziquinone (**39**), with antiparasitic activity were identified in *Zanthoxylum heitzii* (Rutaceae) bark extract. Compound **37** is the most active compound with an IC_50_ value of 0.0089 *μ*g/ml (25 nM), though it is slower in action, while compounds **38** and **39** are less potent, with IC_50_ values of 9.7 ± 1.6 *μ*M and 8.8 ± 0.5 *μ*M, but they are are fast-acting [36].

**Figure 5:**
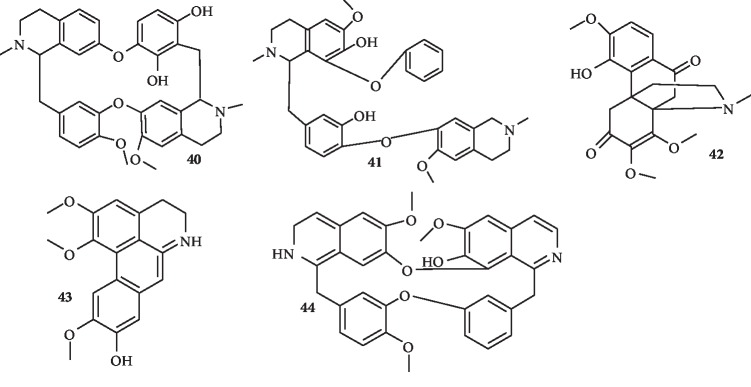
Structures of bisbenzylisoquinoline (**40, 41,** and **44**) and hasubanane (**42** and **43**) alkaloids.

The structures of bisbenzylisoquinoline and hasubanane alkaloids with antiplasmodial activity are shown in Figure 5.

From the leaves of *Stephania abyssinica*, two bisbenzylisoquinoline alkaloids, (−)-pseudocurine (**40**) and (−)-pseudoisocurine (**41**), and one hasubanane alkaloid, (−)-10-oxoaknadinine (**42**), were isolated. Compound **40** exhibits strong antiplasmodial activity against both chloroquine-susceptible D6 and resistant W2 strains of *P. falciparum* (IC_50_ 0.29 ± 0.00 and 0.31 ± 0.01 *μ*g/ml, respectively). Compound **41** shows moderate and mild activity (0.75 ± 0.11 and 1.65 ± 0.03 *μ*g/ml, respectively) and compound **42** shows mild activity against W2 (IC_50_ = 3.45 *μ*g/ml) but was inactive against D6 (IC_50_ = 10.25 *μ*g/ml) [37]. Also, six alkaloids were isolated from *Alseodaphne corneri.* These include (+)-laurotetanine (**43**) and (+)-norstephasubine (**44**), both of which exhibit strong antiplasmodial activity with IC_50_ values of 0.189 and 0.116 *μ*M, respectively [38].

### 1.5. Naphthoisoquinoline Alkaloids

**Figure 6:**
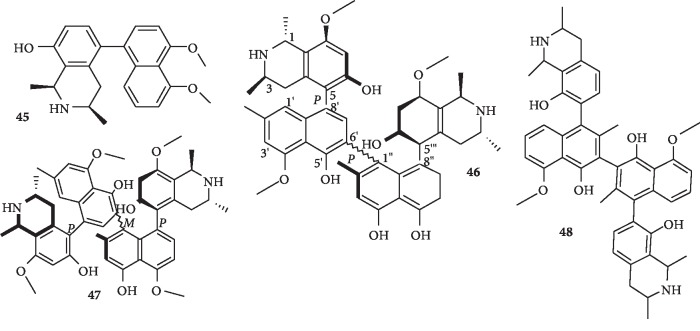
Structures of naphthylisoquinoline (**45**) and dimeric naphthylisoquinoline (**46**–**48-**) alkaloids.

Naphthoisoquinoline alkaloids have also been reported recently, many of which are dimeric. Naphthoisoquinoline alkaloids are usually characterized by the C5/C8′ linkage between the naphthalene and the isoquinoline portions of these alkaloids. Dioncophyllaceous alkaloids possess naphthoisoquinoline nucleus. Dioncophylline F (**45**), the first 5,8′-coupled dioncophyllaceous alkaloid (i.e., lacking an oxygen function at C-6 and possessing an *R*-configuration at C-3), was isolated from Congolese liana *Ancistrocladus ileboensis* with some other alkaloids. The alkaloids show high and specific activities against *P. falciparum* [39]. In addition, dimeric naphthoisoquinoline alkaloids were reported from the Ancistrocladus species. Mbandakamines A (**46**) and B (**47**) were isolated from the leaves of a Congolese Ancistrocladus species as the first dimeric naphthylisoquinoline alkaloids with an unsymmetrically coupled central biaryl axis (Figure 6). Mbandakamine A (**46**) exhibits good antimalarial activity [40].

In a related study on the root bark of the same plant, Ancistrocladus species from the Democratic Republic of Congo, a new dimeric naphthylisoquinoline alkaloid, jozimine A2(**48**), was isolated. Compound **48** is one of the as yet very rare naphthylisoquinoline dimers whose central biaryl axis is rotationally hindered. Moreover, it is the first natural dimer of a dioncophyllaceae-type alkaloid that is lacking oxygen functions at C6 and bearing *R* configurations at C3 in its two isoquinoline portions. The new dimer (**48**) exhibits excellent, and specific, antiplasmodial activity [41].

### 1.6. Aporphine and Morphinandienone Alkaloids

**Figure 7:**
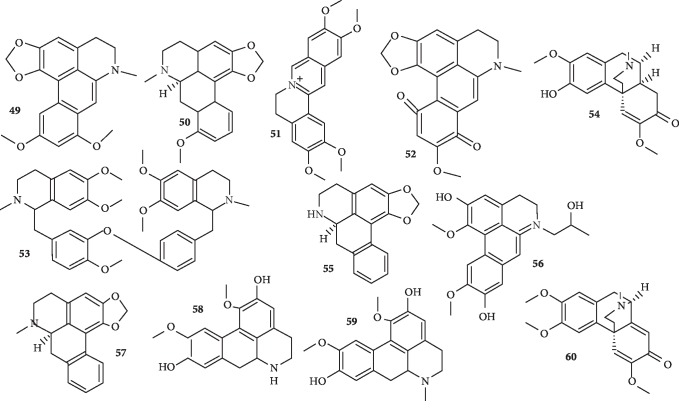
Structures of apophine (**49, 50,** and **52**), isoquinoline (**51**), bisbenzylisoquinoline (**53**), and morphinandienone (**54** and **60**) alkaloids.

Chemically, aporphine alkaloids possess a tetracyclic framework. They incorporate a tetrahydroisoquinoline substructure and belong to the isoquinoline class of alkaloids. Several aporphine alkaloids possessing antimalarial activity have been isolated recently (Figure 7).

A new aporphine alkaloid named vireakine (**49**) along with two known alkaloids, stephanine (**50**) and pseudopalmatine (**51**), described for the first time in *Stephania rotunda*, together with five known alkaloids, was isolated and identified. The compounds were evaluated for their *in vitro* antiplasmodial and cytotoxic activities. All tested compounds show significant antiplasmodial activity with IC_50_ values ranging from 1.2 to 52.3 *μ*M with a good selectivity index for compound **51** with IC_50_ value of 2.8 *μ*M against W2 strain of *P. falciparum* and with IC_50_ > 25 *μ*M on K562S cells [42]. Further, from the twigs of *Dasymaschalon obtusipetalum*, one new p-quinonoid aporphine alkaloid, obtusipetadione (**52**), and eleven known compounds were isolated. Compound **52** shows significant *in vitro* antiplasmodial activity against the *P. falciparum* strains TM4 and K1 (multidrug-resistant strain) with IC_50_ values of 2.46 ± 0.12 and 1.38 ± 0.99 *μ*g/mL, respectively, with no cytotoxicity [43]. In another report, six alkaloids were isolated from the bark of *Dehaasia longipedicellata*. All the compounds display potent-to-moderate activity against the growth of *P. falciparum* K1 isolate (resistant strain) with IC_50_ values ranging from 0.031 to 30.40 *μ*M. Two of the compounds, a bisbenzylisoquinoline, (-)-*O*, *O′*-dimethylgrisabine (**53**), and a morphinadienone, (-)-milonine (**54**), were the most potent compounds, with IC_50_ values of 0.031 and 0.097 *μ*M, respectively, which are comparable to the standard, chloroquine (0.090 *μ*M). All of the compounds showed no potency against lung (A549) cancer cells and weak cytotoxicity against skin (A375) cancer cells [44]. Further, two aporphine alkaloids and other compounds were isolated from *Xylopia sericea* leaves. One of the aporphine alkaloids, anonaine (**55**), shows significant antiplasmodial effect against chloroquine-resistant W2 strain *P. falciparum* and moderate cytotoxicity against HepG2 cells (IC_50_ 23.2 ± 2.7 *μ*g/ml, CC_50_ 38.3 ± 2.3 *μ*g/ml, SI 1.6) [45]. Also, one new aporphine named tavoyanine A (**56**), along with four known aporphines, three of which are roemerine (**57**), laurolitsine (**58**), and boldine (**59**), and one morphinandienone type, sebiferine (**60**), were isolated from the leaves of *Phoebe tavoyana* (Meissn.) Hook f. (Lauraceae). Compounds **56**–**60** were found to exhibit potent inhibitory activity against the growth of *P. falciparum* 3D7 clone, with IC_50_ values of 0.89, 1.49, 1.65, and 2.76 *μ*g/ml, respectively [46]. Compound **60** was previously isolated from *Dehaasia longipedicellata* but was found to have moderate activity against *P. falciparum* KI isolate (resistant strain) with IC_50_ value of 22.46 *μ*M [44].

### 1.7. Protoberberine Alkaloids

**Figure 8:**
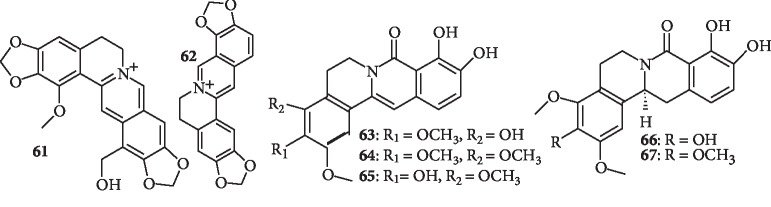
Structures of protoberberine alkaloids.

This class of compounds is mainly found in the plants of the Papaveraceae family. The alkaloids are characterized by a tetracyclic ring system that “hides” a substituted phenethylamine or vanillylamine, depending on the viewing “angle” (Figure 8).

Wangchuk et al. [47] carried out phytochemical studies of the aerial parts of *Meconopsis simplicifolia* (D. Don) Walpers (Papaveraceae), resulting in the isolation of one new protoberberine-type alkaloid, simplicifolianine (**61**), and five known alkaloids. Compound **61** displays the most potent antiplasmodial activity against the *P. falciparum* strains, TM4/8.2 (chloroquine-antifolate-sensitive strain) and K1CB1 (multidrug-resistant strain) with IC_50_ values of 0.78 *μ*g/mL and 1.29 *μ*g/mL, respectively. The compounds did not show any significant cytotoxic activity against carcinoma KB cells and normal Vero cells. In another study, coptisine (**62**), an alkaloid from *Coptidis rhizoma* (Ranunculaceae), was found to be a novel and potent inhibitor of *Plasmodium falciparum* dihydroorotate dehydrogenase (PfDHODH) with an IC_50_ value of 1.83 ± 0.08 *μ*M [48]. Besides, Promchai et al. [49] isolated five new oxoprotoberberine alkaloids, miliusacunines A–E (**63–67**), along with nine known compounds from the leaves and twigs of *Miliusa cuneata* (Annonaceae). All isolated compounds were evaluated for their cytotoxicity against the KB and Vero cell lines and for antimalarial activities against the *P. falciparum* strains TM4 and K1 (sensitive and multidrug-resistant strains, respectively). Compound **63** exhibits *in vitro* antimalarial activity against the TM4 strain, with an IC_50_ value of 19.3 ± 3.4 *μ*M, while compound **64** demonstrates significant activity against the K1 strain, with an IC_50_ value of 10.8 ± 4.1 *μ*M. Both compounds show no discernible cytotoxicity to the Vero cell line at the concentration levels evaluated. In another study, a bioassay-guided fractionation of the tubers of *Stephania venosa* (Blum) Spreng (Menispermaceae) led to the isolation of four aporphine and one tetrahydroprotoberberine alkaloids including stephanine (**50**), which was observed to be the most active antiplasmodial compound but is also the most cytotoxic with the lowest selectivity index [50]. Compound **50** was previously reported in *Stephania rotunda* with significant antiplasmodial activity [42].

### 1.8. Amaryllidaceae Alkaloids

**Figure 9:**
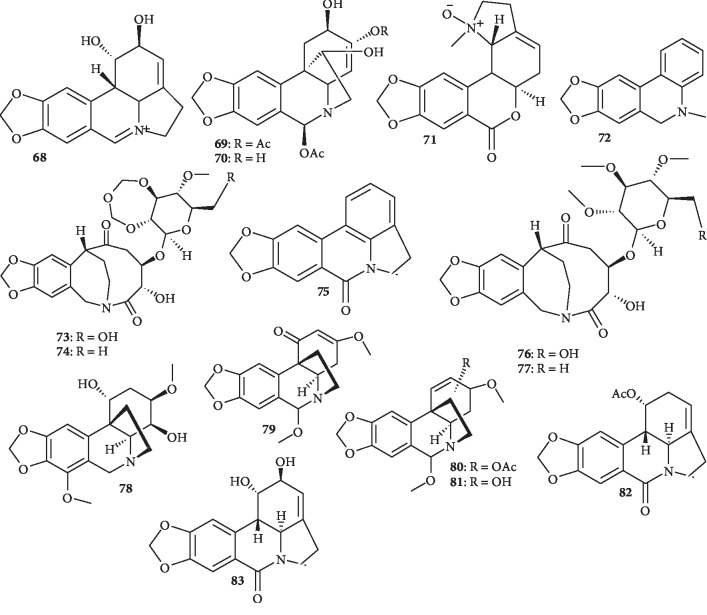
Structures of Amaryllidaceae alkaloids.

A particular characteristic of the family of Amaryllidaceae is a consistent presence of an exclusive group of alkaloids, which have been isolated from the plants of all the genera of this family. The Amaryllidaceae alkaloids represent a large and still expanding group of isoquinoline alkaloids, the majority of which are not known to occur in any other family of plants; these alkaloids are considered to be biogenetically related (Figure 9).

Phytochemical investigation of the bulbs of *Lycoris radiata* (Fam: Amaryllidaceae) resulted in the isolation of five new Amaryllidaceae alkaloids: (+)-5,6-dehydrolycorine (**68**), (+)-3*α*,6*β*-diacetyl-bulbispermine (**69**), (+)-3*α*-hydroxy-6*β*-acetyl-bulbispermine (**70**), (+)-8,9-methylenedioxylhomolycorine-N-oxide (**71**), and 5,6-dihydro-5-methyl-2-hydroxyphenanthridine (**72**), together with two known compounds. Compound **68** exhibits antimalarial activity with IC_50_ values of 2.3 *μ*M for D6 strain and 1.9 *μ*M for W2 strain of *P. falciparum.* Compound **68** also shows potent cytotoxicity against astrocytoma and glioma cell lines (CCF-STTG1, CHG-5, SHG-44, and U251), as well as HL-60, SMMC-7721, and W480 cell lines with IC_50_ values of 9.4–11.6 *μ*M [51]. Also, antimalarial bioassay-guided fractionation of the swamp lily *Crinum erubescens* (Amaryllidaceae) led to the isolation of four compounds with potent antiplasmodial activity. They include three known compounds, cripowellin A (**73**), cripowellin B (**74**), and hippadine (**75**), as well as the new compounds cripowellin C (**76**) and D (**77**). The antiplasmodial activities (IC_50_ values) of compounds **73**, **74, 76**, and **77** were determined to be 30 ± 2, 180 ± 20, 26 ± 2, and 260 ± 20 nM, respectively, while compound **75** is inactive. Their antiproliferative IC_50_ values against the A2780 human ovarian cancer cell line were 11.1 ± 0.4, 16.4 ± 0.1, 25 ± 2, and 28 ± 1 nM, respectively. Presence of OH group in the cripowellin backbone (indicated by R in the structures of **73** and **74** or **76** and **77**) seems to enhance antiplasmodial activity [52].

Crinane alkaloids were also isolated from the Amaryllidaceae family. A chemical investigation of *Amaryllis belladonna* Steud. (Amaryllidaceae) bulbs resulted in the isolation of the new crinane alkaloid 1,4-dihydroxy-3-methoxy powellan (**78**), along with the three known crinane alkaloids, distichamine (**79)**, 11-O-acetylambelline (**80**), and ambelline (**81**), as well as the two lycorane alkaloids, acetylcaranine (**82**) and hippadine (**75**). Compund **82** shows the most potent inhibitory activity, with an IC_50_ value of 3.3 ± 0.3 *μ*M, but compound **75** is inactive despite its structural similarity. Slight structural differences of conjugation and substituents in the tetrahydrophenanthridine moiety seem to greatly affect the inhibitory activity. The crinane-type alkaloid (**80)** exhibits weak inhibitory activity (IC_50_, 35 ± 1 *μ*M), whereas **81** shows stronger activity with an IC_50_ value of 7.3 ± 0.3 *μ*M. The difference in the antiplasmodial activity of these two compounds could be explained by the placement of oxy-substitution at C-11, since acetylation of the oxygenated C-11 of the ethanol bridge slightly decreased the inhibitory activity. The new crinane-type alkaloid **78** showed weak inhibitory activity (IC_50_, 37 ± 3 *μ*M). Compound **79** has little inhibitory activity, despite its similarity to **80** and **81**. Only compound **82** shows inhibitory, though very weak, effect against A2780 ovarian cells with an IC_50_ value of 56 ± 1 *μ*M [53]. In a recent study, lycorine (**83**), along with 14 other alkaloids, was isolated from *Worsleya procera* roots. The compound (**83)** exhibits antiplasmodial activity against both sensitive (3D7) and resistant (K1) *P. falciparum* parasite strains with IC_50_ values of 2.5 and 3.1 *μ*M, respectively, and a low cytotoxic profile against human hepatocarcinoma cells (HepG2), with a selectivity index greater than 100 [54].

### 1.9. Cyclopeptide Alkaloids

**Figure 10:**
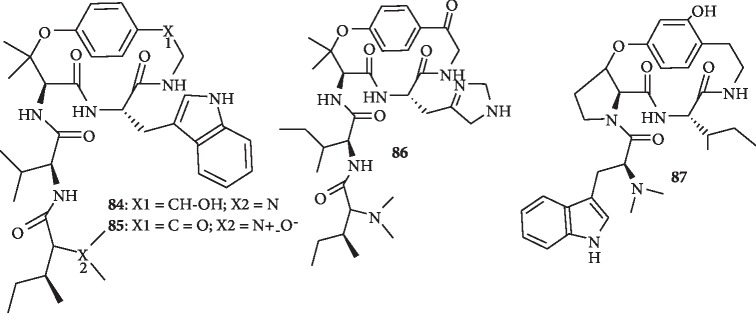
Chemical structures of cyclopeptides with antiplasmodial activity.

Cyclopeptide alkaloids are polyamidic, macrocyclic compounds, containing a 13-, 14-, or 15-membered ring. The ring system consists of a hydroxystyrylamine moiety, an amino acid, and a *β*-hydroxy amino acid; attached to the ring is a side chain, comprised of one or two more amino acid moieties [55]. The structures of the cyclopeptide alkaloids from plants that possess antimalarial activity are shown in Figure 10.

Four cyclopeptide alkaloids were isolated from the root bark of *Hymenocardia acida*. These include hymenocardinol (**84**) as well as hymenocardine *N*-oxide (**85**) and a new cyclopeptide alkaloid containing an unusual histidine moiety named hymenocardine-H (**86**). All the compounds show moderate antiplasmodial activity, with IC_50_ values ranging from 12.2 to 27.9 *μ*M, the most active one being compound **85**, with an IC_50_ value of 12.2 ± 6.6 *μ*M. Compounds **84**–**86** were found not to be cytotoxic against MRC-5 cells (IC_50_ > 64.0 *μ*M) [56]. In addition, nine cyclopeptide alkaloids were isolated from the roots of *Ziziphus oxyphylla*. The most potent compound, *O*-desmethylnummularine-R (**87**), exhibits antiplasmodial activity with an IC_50_ value of 3.2 ± 2.6 *μ*M against *P. falciparum* K1 and cytotoxic activity (IC_50_ value of >64.0 *μ*M) against MRC-5 cells [57].

### 1.10. Quinoline Alkaloids

Chemical investigation of the roots of the Australian desert plant *Eremophila microtheca* yielded a novel quinoline-serrula alkaloid, microthecaline A (**88**), which displays moderate antimalarial activity against *P. falciparum* (3D7 strain), with an IC_50_ value of 7.7 *μ*M [58]. In another study, seven known furoquinoline alkaloids and two known methoxyflavones were isolated from *Melicope madagascariensis* (Rutaceae). One of the compounds, 6-methoxy-7-hydroxydictamnine (heliparvifoline) (**89**), exhibits weak antimalarial activity (IC_50_ = 35 *μ*M) against the chloroquine-resistant strain Dd2 of *P. falciparum* [59].

### 1.11. Pyridocoumarin Alkaloids

Chemical investigation of the aerial parts of *Goniothalamus australis* resulted in the isolation of two pyridocoumarin alkaloids, goniothalines A (**90**) and B (**91**), as well as eight known natural products, including sauristolactam (**92**), an aristolactam alkaloid, as well as (-)-anonaine (**55**). Compounds **92** and **55** are active against a chloroquine-sensitive *P. falciparum* line (3D7) with IC_50_ values of 9.0 and 7.0 *μ*M, respectively. The novel natural products (**90** and **91**) display no *in vitro* antiparasitic activity at 50 *μ*M. Data from the study suggested that 3-methoxy substituent moderately reduces biological function. Also methylation of the nitrogen and demethoxylation at C-8 in the aristolactam skeleton (compound **92**) are important for *P. falciparum* growth inhibition [60].

### 1.12. Acridone Alkaloids

The root bark of *Zanthoxylum simullans* Hance afforded five known acridone alkaloids, together with other compounds. Their antimalarial activity was tested against two different strains of the parasite *P. falciparum*, 3D7 and Dd2. Normelicopidine (**93**) is the most active against Dd2 with IC_50_ value of 18.9 ug/mL [61].

### 1.13. Macrocyclic Alkaloids

The macrocyclic dilactone alkaloid, carpaine (**94**), was isolated from *Carica papaya* L. leaf. The compound (**94)** was screened against *Plasmodium falciparum* 3D7 and Dd2 strains. The cytotoxicity was evaluated against NL20 cells. The compound exhibits good activity against both strains of *P. falciparum*, 3D7 and Dd2, with IC_50_ values of 4.21 *μ*M and 4.57 *μ*M, respectively, and high selectivity for the parasite and was nontoxic to healthy uninfected human red blood cells [62]. The effects of **94** may be related to its macrocyclic dilactone structure, a possible cation chelating structure.

**Figure 11:**
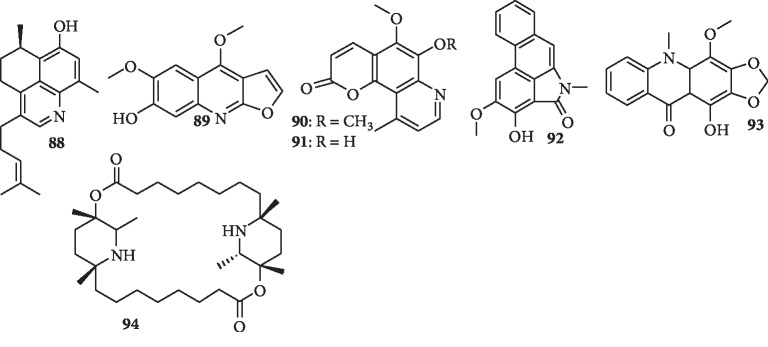
Chemical structures of quinoline (**88** and **89**), pyridocoumarin (**90** and **91**), aristolactam (**92**), acridone (**93**), and macrocytic (**94**) alkaloids with antiplasmodial activity.

The chemical structures of the quinoline, pyridocoumarin, aristolactam, acridone, and macrocytic alkaloids are shown in Figure 11.  Table 1 presents the summary of the alkaloids and their antimalarial activity.

## 2. Conclusion

In the present review, attempt has been made to document the alkaloidal compounds isolated from medicinal plants and/or investigated recently for their antimalarial property. They belong to a great structural diversity including terpenoide, steroid, indole, phenanthroindolizine, isoquinoline, benzylisoquinoline, hasubanane, naphthoisoquinoline, aporphine, morphinandienone, protoberberine, Amaryllidaceae, cyclopeptide, quinoline, pyridocoumarin, acridone, and macrocyclic alkaloids. Many of the compounds with interesting antimalarial property were reported for the first time within this period and they present an array of potential lead compounds towards development of novel antimalarial drugs. Their antiplasmodial and cytotoxicity data were presented. Structure activity relationships were discussed where possible.

Particularly, the most interesting of the newly isolated compounds include the cassane-type diterpene alkaloids, caesalminines A (**1**) and caesalminines B (**2**), and the bisindole alkaloid, strychnobaillonine (**19**). Some other known compounds, including the isoquinolines, cycleanine (**25**), and 10-demethylxylopinine (**26**), were also reported to show strong antimalarial property with low IC_50_ values. However, some of the compounds have relatively high cytotoxicity profile, while some have not been investigated for their toxicity profile. Another observation is that majority of the study of the antimalarial activity has been based on *in vitro* studies, while *in vivo* studies are quite rare.

Given the above, it is suggested that further structural modifications and structure-activity relationship study should be done on the promising compounds. The identified alkaloids with promising antimalarial activity should serve as lead compounds for drug design to obtain compounds with improved efficacy and lower toxicity. They should also be subjected to toxicity and *in vivo* efficacy studies. Additionally, the mode of action of the promising compounds should be explored. It is hoped that the outcome of the present review will spur further research into the structural modification and/or development of the promising compounds as novel antimalarial drugs.

## Figures and Tables

**Table 1:** Summary of antimalarial alkaloids derived from plants.

Name	Class of alkaloid	Source	Activity against *P. falciparum*	Reference
Two new compounds: caesalminines A (**1**) and caesalminines B (**2**)	Cassane-type diterpene alkaloids	Seeds of *Caesalpinia minax*	*In vitro* activity with IC_50_: 0.42 *μ*M (**1**) and 0.79 *μ*M (**2**)	[21]

Three new compounds: 8*α*-polyveolinone (**3**), *N*-acetyl-8*α*-polyveolinone (**4**), and *N*-acetyl-polyveoline (**5**)	Indolosesquiterpene alkaloids	Stem bark of *Polyalthia oliveri*	Compounds **4** and **5** exhibit moderate activity against NF54 strain, with low cytotoxicity on L6 cell line	[22]

Three known alkaloids together with strychnochrysine (**6**)	Bisindolomonoterpenic alkaloid	*Strychnos nux-vomica* L	Compound **7** is the most active with IC_50_ 10 *μ*M against D7 strain	[23]

Two known alkaloids: polyalthenol (**7**) and *N*-acetyl-polyveoline (**5**), together with other compounds	Indolosesquiterpene	*Greenwayodendron suaveolens* (Engl. & Diels) Verdc. (syn. *Polyalthia suaveolens* Engl. & Diels)	Highest activity was found for compound **5** against K1 strain with IC_50_ value of 2.8 *μ*M, SI = 10.9	[24]

Conesine (**8**)	Steroidal alkaloid	*Holarrhena antidysenterica*	*Shows in vivo acivity against P. berghei* Also shows *in vitro* activity against *P. falciparum* with IC₅₀ values of 1.9 *μ*g/ml and 1.3 *μ*g/ml in the schizont maturation and pLDH assays, respectively; cytotoxicity IC_50_ of 14 *μ*g/ml	[25]

New compound: mokluangin D (**9**) with nine known steroidal alkaloids including irehline (**10**) and mokluangin A (**11**)	Pregnene-type alkaloid	*Holarrhena pubescens* roots	Compounds **10** and **11** show strong activity against K1 strain with IC_50_ values of 1.2 and 2.0 *μ*M and weak cytotoxicity against the NCI-H187 cell line	[26]

The known alkaloid, *N*-3-benzoyldihydrocyclomicrophylline F (**12**), together with other compounds	Buxus alkaloid (a steroidal alkaloid) (compound **12**)	*Buxus cochinchinensis* Pierre ex Gagnep.	Compound **12** is active with IC_50_ value of 2.07 ± 0.13 *μ*M; it shows cytotoxicity with IC_50_ value of 1.9 *μ*M (against HT-29 human carcinoma) and > 20 (against NF-KB)	[27]

Three new compounds: alstoniaphyllines A **(13)**, alstoniaphyllines B (**14**), and alstoniaphylline C (**15**), and eight known alkaloids including alstonisine (**16**)	Nitrogenous derivatives (**13** and **14**) and indole alkaloid (**15**)	*Alstonia macrophylla* bark	Compound **16** exhibits activity with IC_50_ value of 7.6 *μ*M	[28]

A new compound, 12-hydroxy-*N*-acetyl-21(*N*)-dehydroplumeran-18-oic acid (**17**), and 11 known indole alkaloids including 20-epi-dasycarpidone (**18**)	Indole alkaloid	*Aspidosperma ulei* Markgr	Only compound **18** is active (IC_50_ value of 16.7 mM) against K1 strain and shows no toxicity to fibroblasts (IC_50_ > 50 mg/mL).	[29]

A new compound: strychnobaillonine (**19**)	Bisindole	Roots of *Strychnos icaja*	Shows *in vitro* activity with IC_50_ value of 1.1 *μ*M	[30]

Two new alkaloids together with five known alkaloids, including 16-demethoxycarbonylvoacamine (**20**)	Sarpagine-type indole alkaloids	Bark of *Tabernaemontana macrocarpa* Jack	Compound **20** shows activity against *P. falciparum* 3D7 and cytotoxic activity against human cell line, HepG2 cells	[31]

New alkaloid, 4a,b-*seco*-dehydroantofine (**21**), and three known alkaloids, dehydrotylophorine (**22**), dehydroantofine (**23**), and tylophoridicine D (**24**)	Phenanthroindolizine alkaloids	Twigs of *Ficus septica*	Compound **21** displays moderate activity against the 3D7 strain with IC_50_ value of 4.0 *μ*M, whereas the known alkaloids **22**–**24** display strong activity (IC_50_ 0.028, 0.42, and 0.058 *μ*M, respectively). The cytotoxicities of the compounds (**21**–**24)** against L929 cells are in the range of 8.2–56 *μ*M, while selectivity index is in the range of 14.0–1964.0	[32]

Seven known alkaloids: cycleanine (**25**), 10-demethylxylopinine(**26**), reticuline (**27**), laurotetanine (**28**), bicuculine (**29**), *α*-hydrastine (**30**), and anolobine (**31**)	Isoquinoline alkaloids	Bark of *Actinodaphne macrophylla*	They show *in vitro* activity with IC_50_ values of 0.08 *μ*M, 0.05 *μ*M, 1.18 *μ*M, 3.11 *μ*M, 0.65 *μ*M, 0.26 *μ*M, and 1.38 *μ*M, respectively, against 3D7 strain	[33]

Four known alkaloids: (+)-*N*-methylisococlaurine (**32**), atherosperminine (**33**), 2-hydroxyathersperminine (**34**), and noratherosperminine (**35**)	Isoquinoline alkaloids	stem bark of *Cryptocarya nigra*	They display activity with IC_50_ values of 5.40, 5.80, and 0.75 *μ*M, respectively, for compounds **32**, **33**, and **34**	[34]

Four alkaloids including palmatine (**36**)	Isoquinoline alkaloids	Leaves of *Annickia kummeriae*	Compound **36** exhibits the highest activity against *P. falciparum* K1 (IC_50_ 0.080 ± 0.001 *μ*g/mL, SI = 1154)	[35]

Known compounds: dihydronitidine (**37**), pellitorine (**38**), and heitziquinone (**39**)	Isoquinoline (dihydronitidine), decadienamide (pellitorine), and benzophenthrathridine (heitziquinone)	*Zanthoxylum heitzii* bark	Dihydronitidine (**37**) is the most active compound with an IC_50_ value of 25 nM, while compounds **38** and **39** have IC_50_ values of 9.7 ± 1.6 *μ*M and 8.8 ± 0.5 *μ*M, respectively	[36]

Three new alkaloids, (-)-pseudocurine (**40**), (-)-pseudoisocurine (**41**), and (-)-10-oxoaknadinine (**42**)	Bisbenzylisoquinoline alkaloids (**40** and **41**) and one hasubanane alkaloid (**42**)	Leaf of *Stephania abyssinica*	They show strong-to-mild activity with IC_50_ values ranging from 0.29 ± 0.00 to 1.65 ± 0.03 *μ*g/mL against both chloroquine-susceptible D6 and chloroquine-resistant strains	[37]

Six known alkaloids including (+)-laurotetanine (**43**) and (+)-norstephasubine (**44**)	Bisbenzylisoquinoline	*Alseodaphne corneri*	Both compounds **43** and **44** show strong activity with IC_50_ values of 0.189 and 0.116 *μ*M, respectively, against K1 strain and no cytotoxicity against hTERT-HPNE cell line	[38]

One new dioncophyllaceous alkaloid, dioncophylline F (**45**), and other known alkaloids	Naphthylisoquinoline alkaloid (compound **45**)	*Ancistrocladus ileboensis*	Compound **45** together with others shows high and specific activities against *P. falciparum*	[39]

New compounds: mbandakamines A (**46**) and B (**47**)	Dimeric naphthylisoquinoline alkaloids with an unsymmetrically coupled central biaryl axis	Unidentified *Congolese Ancistrocladus* species	Compound **46** exhibits good antimalarial activity	[40]

New compound: jozimine A2 (**48**)	Dimeric naphthylisoquinoline alkaloid	Ancistrocladus species	Compound **48** exhibits excellent and specific antiplasmodial activity	[41]

New alkaloid, vireakine (**49**), along with other known alkaloids including stephanine (**50**) and pseudopalmatine (**51**)	Aporphine alkaloid (**49**)	Tuber of *Stephania rotunda*	Activity ranged from 1.2 *μ*M to 52.3 *μ*M for all tested compounds; compound **51** (IC_50_ = 2.8 *μ*M) shows good selectivity index against W2 chloroquine-resistant strain	[42]

A new alkaloid, obtusipetadione (**52**), and eleven known compounds	p-Quinonoid aporphine alkaloid (**52**)	Twigs of *Dasymaschalon obtusipetalum*	Compound **52** is active with IC_50_ values of 2.46 ± 0.12 and 1.38 ± 0.99 *μ*g/mL, respectively, for TM4 and K1 strains with no cytotoxicity	[43]

Several known alkaloids including (-)-O-O-dimethylgrisabine (**53**) and (-)-milonine (**54)**	Morphinandienones; aporphines and benzlyisoquinoline	Bark of of *Dehaasia longipedicellata*	They display potent-to-moderate activity with IC_50_ values ranging from 0.031 to 30.40 *μ*M. Compounds **53** and **54** are the two most potent compounds, with IC_50_ values of 0.031 and 0.097 *μ*M, respectively, against K1 strain. All the compounds show no potency against lung (A549) cancer cells	[44]

A new alkaloid, anonaine (**55**), and other alkaloids	Aporphine alkaloids	*Xylopia sericea* leaves	Compound **55** is active against chloroquine-resistant W2 strain *P. falciparum* (IC_50_ 23.2 ± 2.7 *μ*g/mL) and has moderate cytotoxicity against HepG2 cells (SI = 1.6)	[45]

A new alkaloid, tavoyanine A (**56**), and other known alkaloids, roemerine (**57**), laurolitsine (**58**), boldine (**59**), and sebiferine (**60**)	Aporphine alkaloids (**56**–**59**) and morphinandienone (**60**)	Leaf of *Phoebe tavoyana*	Compounds **56**–**60** are active against *P. falciparum* with IC_50_ values of 0.89, 1.49, 1.65, and 2.76 *μ*g/mL, respectively	[46]

A new alkaloid, simplicifolianine (**61),** together with five known alkaloids	Protoberberine-type alkaloid	Aerial components of *Meconopsis simplicifolia* (D. Don) Walpers	Compound **22** is the most potent against TM4/8.2 and K1CB1 with IC_50_ values of 0.78 *μ*g/mL and 1.29 *μ*g/mL, respectively	[47]

A known alkaloid, coptisine (**62**)	Protoberberine	*Coptidis rhizoma*	Compound **62** is found to be an inhibitor of PfDHODH with an IC_50_ value of 1.83 ± 0.08 *μ*m	[48]

Five new alkaloids, miliusacunines A-E (**63**–**67**), along with nine known compounds	Oxoprotoberberine alkaloids	Leaf and twigs of *Miliusa cuneata*	Compound **63** shows activity against the TM4 strain (IC_50_ 19.3 ± 3.4 *μ*M) and compound **64** shows activity against the K1 strain (IC_50_ 10.8 ± 4.1 *μ*M). Both show no cytotoxicity to Vero cells	[49]

One tetrahydroprotoberberine and four aporphine alkaloids including stephanine (**50**)	Tetrahydroprotoberberine alkaloid (compound **50**)	Tubers of *Stephania venosa* (Blum) Spreng	Stephanine (**50**) is the most interesting but is the most cytotoxic with the lowest selectivity index	[50]

Five new alkaloids, (+)-5,6-dehydrolycorine (**68**), (+)-3*α*,6*β*-diacetyl-bulbispermine (**69**), (+)-3*α*-hydroxy-6*β*-acetyl-bulbispermine (**70**), (+)-8,9-methylenedioxylhomolycorine-N-oxide (**71**), and 5,6-dihydro-5-methyl-2-hydroxyphenanthridine (**72**), together with other known compounds	Amaryllidaceae alkaloids	Bulbs of *Lycoris radiata*	Compound **68** is active with IC_50_ values of 2.3 *μ*M for D6 strain and 1.9 *μ*M for W2 strain; compound **68** also shows cytotoxicity against various carcinoma cells with IC_50_ values of 9.4–11.6 *μ*M	[51]

Three known alkaloids, cripowellin A (**73**), cripowellin B (**74**), and hippadine (**75**), as well as the new compounds cripowellin C (**76**) and D (**77**)	Cripowellin alkaloids	*Crinum erubescens*	Compounds **73**, **74, 76**, and **77** are active with IC_50_ values of 30 ± 2, 180 ± 20, 26 ± 2, and 260 ± 20 nM, respectively, while **75** is inactive	[52]

New alkaloid, 1,4-dihydroxy-3-methoxy powellan **(78)**, along with the known alkaloids, distichamine (**79)**, 11-O-acetylambelline (**80**), ambelline (**81**), acetylcaranine (**82**), and hippadine (**75**)	Crinane (**78**–**81**) and lycorane (**82** and **75**) alkaloids	*Amaryllis belladonna* Steud. Bulbs	Acetylcaranine (**82**) exhibits strong activity (IC_50_ value of 3.3 ± 0.3 *μ*M), while compound **78** is inactive. Compound **82** shows weak inhibition against A2780 ovarian cells with an IC_50_ value of 56 ± 1 *μ*M	[53]

Lycorine (**83**), along with 14 other alkaloids	Amaryllidaceae alkaloids	*Worsleya procera* roots	Compound **83** was active against both sensitive (3D7) and resistant (K1) *P. falciparum* strains with IC_50_ values of 2.5 and 3.1 *μ*M, respectively, and a low cytotoxicity against HepG2 cells	[54]

Three new alkaloids, hymenocardinol (**84**), hymenocardine *N*-oxide (**85**), and hymenocardine-H (**86**), and one known alkaloid	Cyclopeptide alkaloids	Root bark of *Hymenocardia acida*	All compounds show moderate activity with IC_50_ values ranging from 12.2 to 27.9 *μ*M, the most active being compound **85** (IC_50_ 12.2 ± 6.6 *μ*M). They are not cytotoxic against MRC-5 cells (IC_50_ > 64.0 *μ*M)	[56]

Nine alkaloids including *O*-desmethylnummularine-R (**87**)	Cyclopeptide alkaloids	Roots of *Ziziphus oxyphylla*	Most promising activity is found for compound **87** with IC_50_ value of 3.2 ± 2.6 *μ*M against K1 strain and cytotoxcity (IC_50_ value of >64.0 *μ*M) against MRC-5 cells	[57]

A new alkaloid, microthecaline A (**88**)	Quinoline-serrulatane	*Eremophila microtheca*	Compound **88** exhibits moderate activity against *P. falciparum* (3D7 strain), with an IC_50_ value of 7.7 *μ*M	[58]

Seven known alkaloids including 6-methoxy-7-hydroxydictamnine (heliparvifoline) (**89**) and two known compounds	Furoquinoline alkaloids	Melicope madagascariensis	Heliparvifoline (**89**) shows weak antimalarial activity (IC_50_ = 35 *μ*M) against Dd2 strain	[59]
Two new alkaloids, goniothalines A (**90)** and B (**91**), as well as eight known compounds including sauristolactam (**92**) as well as anonaine (**55)**	Pyridocoumarin alkaloids	Aerial parts of *Goniothalamus australis*	Sauristolactam (**92**) and (-)-anonaine (**55**) exhibit the most potent activity with IC_50_ values of 9 and 7 *μ*M, respectively	[60]

Known alkaloids including normelicopine (**93**)	Acridone alkaloids	*Zanthoxylum simullans* Hance	Normelicopidine (**93**) is the most active against Dd2 with IC_50_ value of 18.9 *μ*g/mL	[61]

Carpaine (**94**)	Macrocyclic alkaloids	*Carica papaya* L. leaf	The compound is active against both 3D7 and Dd2 strains with IC_50_ values of 4.21 *μ*M and 4.57 *μ*M, respectively, and high selectivity for the parasite	[62]
